# Survival benefit of thermal ablation therapy for patients with stage II-III non-small cell lung cancer: A propensity-matched analysis

**DOI:** 10.3389/fonc.2022.984932

**Published:** 2022-08-23

**Authors:** Wei-Yu Yang, Yu He, Qikang Hu, Muyun Peng, Zhe Zhang, Shouzhi Xie, Fenglei Yu

**Affiliations:** ^1^ Department of Thoracic Surgery, The Second Xiangya Hospital of Central South University, Changsha, China; ^2^ Hunan Key Laboratory of Early Diagnosis and Precise Treatment of Lung Cancer, The Second Xiangya Hospital of Central South University, Changsha, China

**Keywords:** non-small cell lung carcinoma, thermal ablation, survival, stage II-III, SEER

## Abstract

**Background:**

Thermal ablation (TA) is considered a safe alternative to surgical resection for the treatment of non-small cell lung cancer (NSCLC). While previous studies have shown that TA is beneficial for stage I NSCLC patients, however, few have reported on TA efficacy in patients with stage II-III NSCLC. The current study investigated the impact of TA on the overall survival (OS) and cancer-specific survival (CSS) of patients with stage II-III NSCLC.

**Methods:**

Data on patients with stage II-III NSCLC who did not undergo surgical resection between 2004 and 2015 were extracted from the Surveillance, Epidemiology, and End Results (SEER) database. Propensity score matching (PSM), Kaplan-Meier survival curves, and Cox regression were used for statistical analyses.

**Results:**

A total of 57,959 stage II-III NSCLC patients who did not undergo surgical resection were included in this study, 261 of whom received TA. Overall, TA was associated with a longer OS (*p* = 0.035) and CSS (*p* = 0.005) than non-ablation. After 1:3 PSM, 252 patients receiving TA and 732 patients not receiving ablation were enrolled in the matched cohort. The OS (*p* = 0.047) and CSS (*p* = 0.029) remained higher in the TA group than in the non-ablation group after PSM. Cox regression analysis showed that age, sex, primary tumor site, pathological type, tumor size, radiotherapy, chemotherapy, and thermal ablation were independently associated with OS and CSS (*p <*0.05). Subgroup analysis found that the advantages of TA were more pronounced among individuals ≥70 years of age, with tumor size ≤3.0 cm, or who did not receive radiotherapy.

**Conclusion:**

TA could be an effective alternative treatment for stage II-III NSCLC patients unsuitable for surgical resection, particularly those ≥70 years of age, with tumor size ≤3.0 cm, or who have not received radiotherapy.

## Introduction

Lung cancer, one of the most prevalent and lethal malignancies worldwide, is a significant public health concern (1). In the United States, it is estimated that 235,760 new cases of lung cancer will be diagnosed in 2021, and 131,800 patients will die from the disease (2). Surgical resection is the preferred treatment for early-stage non-small cell lung cancer (NSCLC) (3, 4). However, a proportion of NSCLC patients are unsuitable for surgical resection for several reasons including advanced age, severe underlying disease, and refusal to undergo surgical intervention (5, 6). Thus, non-surgical treatment for NSCLC, including thermal ablation (TA) and stereotactic body radiotherapy (SBRT), has attracted increasing attention (7–11).

TA includes radiofrequency ablation (RFA), microwave ablation (MWA), and ultrasound ablation using various energy sources (12, 13). As a minimally invasive treatment technique, TA utilizes thermal energy to cause injuries to the targeted tissue (14). Of the ablation technologies in clinical use, RFA is the most widely used for the treatment of multiple conditions, including cardiac arrhythmias and liver, lung, kidney, and other tumors (12). MWA devices use higher frequency electromagnetic waves than RFA, allowing larger tissue volumes to be heated (13). Thermal injuries caused by TA may result in protein denaturation, enzyme inactivation, vascular injury, and ischemia-reperfusion, leading to irreversible cellular damage (15, 16). Previous studies indicate that TA is beneficial for stage I NSCLC patients (7, 17–19). Current guidelines from multiple societies specify that the best candidates for TA are stage I NSCLC patients who are not eligible for or amenable to surgical resection and SBRT (20, 21). However, few studies have reported on TA efficacy in patients with stage II-III NSCLC. Thus, it remains unknown whether inoperable stage II-III NSCLC patients may benefit from this technique.

According to the American Society of Clinical Oncology and Chinese Medical Association guidelines, inoperable stage III NSCLC patients should be offered concurrent chemoradiotherapy as a standard treatment (22, 23). Those who are not candidates for concurrent chemoradiation but are candidates for chemotherapy should be offered both sequential chemotherapy and radiation therapy. For inoperable stage II-III NSCLC patients who are unsuitable for chemoradiation, radiotherapy alone is the standard treatment with a survival benefit (23–25). The survival benefit of TA is comparable to SBRT in stage I NSCLC patients (26, 27). The current study performed a retrospective analysis to investigate whether inoperable stage II-III NSCLC patients can benefit from TA using patient data from the Surveillance, Epidemiology, and End Results (SEER) database.

## Methods

### Data source

Data were retrieved from the SEER 18 Registries Database (2000–2018). SEER is a national population-based registry program that collects the tumor-related clinical data and basic demographics of cancer patients (28). Since this database is publicly available and all records are de-identified, no ethics committee approval or informed consent is required.

### Study population

Data from patients diagnosed with NSCLC during 2004–2015 were extracted from SEER. The third edition of the International Classification of Diseases for Oncology (ICD-O-3) was used to identify NSCLC. Patients were included in the study if they had a pathological diagnosis of NSCLC and their age at diagnosis was >18 years. Patients were excluded if (1) they received other surgical procedures beyond thermal ablation, or surgery information was unknown (2), their tumors were identified as TNM stage I or IV (3), they were identified in autopsy or death certificates (4), their survival data was unknown, or (5) information regarding age, sex, race, pathological type, primary tumor site, laterality, lymph node staging, marital status, or tumor size was missing.

The following information was extracted: year of diagnosis, age at diagnosis, sex, race, histological grade, pathological type, primary tumor site, laterality, lymph node staging, tumor size, radiotherapy, chemotherapy, surgery, marital status, cancer-specific survival (CSS), overall survival (OS), and survival months. TNM staging was reclassified according to the eighth edition of the American Joint Committee on Cancer (AJCC) staging manual. Patients were divided into TA and non-ablation groups based on the treatment they received. The primary endpoints in this study were OS and CSS. CSS was defined as the time from diagnosis to death attributed to NSCLC.

### Propensity score matching

In retrospective cohort studies, treatment-related selection bias resulting from an imbalance in the baseline characteristics is inevitable (29). Propensity score matching (PSM) can reduce the selection bias, offset differing clinical features among groups, and bolster the evidence of a retrospective cohort study (30). The current study created a logistic regression model with propensity scores to balance the baseline characteristics between the TA and non-ablation groups. TA was defined as the dependent variable, while other baseline characteristics were included as the covariables. The PSM was performed in a 1:3 ratio using nearest neighbor matching with a caliper of 0.001. Chi-square or Fisher’s exact tests were used to compare baseline characteristics between groups.

### Statistical analysis

Analyses were performed using R version 4.0.0 and SPSS version 26.0. Kaplan-Meier methods and log-rank tests were used for survival analysis. Univariate and multivariable analyses were performed using Cox proportional hazards regression. Variables with *p* <0.10 from the univariable analysis were considered for multivariable analysis. Differences with *p <*0.05 were considered significant.

## Results

### Baseline characteristics

Data on 57,959 non-surgical patients who were diagnosed with stage II-III NSCLC during 2004–2015 were extracted from the SEER database. A total of 261 patients (0.45%) received TA and 57,698 patients (99.55%) did not. The baseline characteristics of the patients are shown in **Table 1**. There were significant differences in age (*p* = 0.003), race (*p* = 0.013), tumor site (*p <*0.001), pathological type (*p <*0.001), tumor size (*p* = 0.002), lymph node staging (*p* = 0.022) and chemotherapy (*p* = 0.003) between the TA and non-ablation groups in the unmatched cohort. After 1:3 PSM, 252 patients receiving TA and 732 patients without ablation were enrolled in the matched cohort. The baseline characteristics were well-balanced between the TA and non-ablation groups in the matched cohorts.

**Table 1:** Characteristics of stage II-III NSCLC patients before and after PSM, n (%).

Characteristics	Before PSM				After PSM		
	Total	Non-ablation(*N* = 57,698)	Thermal ablation(*N* = 261)	*p* Value	Non-ablation(*N* = 732)	Thermal ablation(*N* = 252)	*p* Value
Age (years)				0.003			0.582
<70	25,216 (43.51)	25,079 (43.47)	137 (52.49)		360 (49.18)	129 (51.19)	
≥70	32,743 (56.49)	32,619 (56.53)	124 (47.51)		372 (50.82)	123 (48.81)	
Sex				0.072			0.621
Male	33,008 (56.95)	32,845 (56.93)	163 (62.45)		463 (63.25)	155 (61.51)	
Female	24,951 (43.05)	24,853 (43.07)	98 (37.55)		269 (36.75)	97 (38.49)	
Race				0.013			0.704
White	46,533 (80.29)	46,306 (80.26)	227 (86.97)		629 (85.93)	219 (86.90)	
Black	7,805 (13.47)	7,778 (13.48)	27 (10.34)		87 (11.89)	26 (10.32)	
Others	3,621 (6.25)	3,614 (6.26)	7 (2.68)		16 (2.19)	7 (2.78)	
Laterality				0.544			0.709
Left	23,719 (40.92)	23,617 (40.93)	102 (39.08)		275 (37.57)	98 (38.89)	
Right	34,240 (59.08)	34,081 (59.07)	159 (60.92)		457 (62.43)	154 (61.11)	
Tumor site				<0.001			0.030
Lung lobe	54,424 (93.90)	54,217 (93.97)	207 (79.31)		639 (87.30)	207 (82.14)	
Main bronchus	3,022 (5.21)	2,970 (5.15)	52 (19.92)		88 (12.02)	45 (17.86)	
Overlapping lesion of lung	513 (0.89)	511 (0.89)	2 (0.77)		5 (0.68)	0 (0.00)	
Histological grade				0.166			0.892
I/II	12,809 (22.10)	12,740 (22.08)	69 (26.44)		196 (26.78)	66 (26.19)	
III/IV	17,887 (30.86)	17,805 (30.86)	82 (31.42)		212 (28.96)	77 (30.56)	
Unknown	27,263 (47.04)	27,153 (47.06)	110 (42.15)		324 (44.26)	109 (43.25)	
Pathological type				<0.001			0.390
Squamous cell carcinoma	27,742 (47.86)	27,568 (47.78)	174 (66.67)		480 (65.57)	165 (65.48)	
Adenocarcinoma	26,197 (45.20)	26,125 (45.28)	72 (27.59)		226 (30.87)	72 (28.57)	
Large cell lung cancer	1,812 (3.13)	1,805 (3.13)	7 (2.68)		11 (1.50)	7 (2.78)	
Others	2,208 (3.81)	2,200 (3.81)	8 (3.07)		15 (2.05)	8 (3.17)	
Tumor size				0.002			0.984
≤3.0 cm	14,297 (24.67)	14,206 (24.62)	91 (34.87)		259 (35.38)	90 (35.71)	
3.1–5.0 cm	18,950 (32.70)	18,871 (32.71)	79 (30.27)		218 (29.78)	74 (29.37)	
5.1–7.0 cm	13,797 (23.80)	13,746 (23.82)	51 (19.54)		145 (19.81)	48 (19.05)	
>7.0 cm	10,915 (18.83)	10,875 (18.85)	40 (15.33)		110 (15.03)	40 (15.87)	
Lymph node staging				0.022			0.992
N0	16,012 (27.63)	15,920 (27.59)	92 (35.25)		257 (35.11)	89 (35.32)	
N1	5,778 (9.97)	5,750 (9.97)	28 (10.73)		77 (10.52)	25 (9.92)	
N2	28,429 (49.05)	28,313 (49.07)	116 (44.44)		331 (45.22)	114 (45.24)	
N3	7,740 (13.35)	7,715 (13.37)	25 (9.58)		67 (9.15)	24 (9.52)	
Radiotherapy				0.883			0.741
Yes	34,901 (60.22)	34,745 (60.22)	156 (59.77)		427 (58.33)	150 (59.52)	
No	23,058 (39.78)	22,953 (39.78)	105 (40.23)		305 (41.67)	102 (40.48)	
Chemotherapy				0.003			0.752
Yes	33,541 (57.87)	33,414 (57.91)	127 (48.66)		357 (48.77)	120 (47.62)	
No	24,418 (42.13)	24,284 (42.09)	134 (51.34)		375 (51.23)	132 (52.38)	
Marital status				0.918			0.802
Married	29,572 (51.02)	29,438 (51.02)	134 (51.34)		368 (50.27)	129 (51.19)	
Not married	28,387 (48.98)	28,260 (48.98)	127 (48.66)		364 (49.73)	123 (48.81)	

NSCLC, non-small cell lung cancer; PSM, propensity score matching.

### Survival analysis

**Figure 1:**
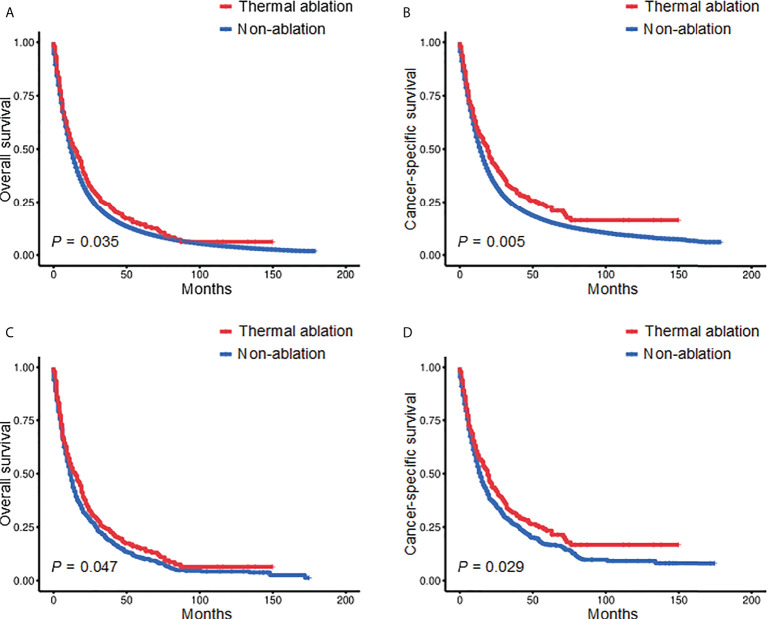
Kaplan-Meier survival curves of stage II-III NSCLC patients before **(A, B)** and after **(C, D)** PSM. NSCLC, non-small cell lung cancer; PSM, propensity score matching.

Kaplan‐Meier analysis revealed a significant difference in the OS of patients receiving TA and those who did not (*p* = 0.035) (**Figure 1A**). The median OS was 15 months (95% confidence interval (CI): 11.275–18.775) and 12 months (95% CI: 11.847–12.153) for the TA and non-ablation groups, respectively. The 3- and 5-year OS of the TA and non-ablation groups was 24.14% versus 18.82% and 14.39% versus 10.84%, respectively. Patients who received TA also had longer CSS than non-ablation patients (*p* = 0.005) (**Figure 1B**). After 1:3 matching, patients who received TA also had longer OS than non-ablation patients (*p* = 0.047) (**Figure 1C**). The median OS was 15 months (95% CI: 11.111–18.889) and 11 months (95% CI: 9.811–12.189) for the TA and non-ablation groups, respectively. The 3- and 5-year OS of the TA and non-ablation groups was 24.21% versus 19.51% and 14.98% versus 10.71%, respectively. CSS was also longer for patients in the TA group than in the non-ablation group (*p* = 0.029) (**Figure 1D**).

### Univariate and multivariate COX regression analysis after PSM

Univariate survival analysis showed that age, sex, race, primary tumor site, histological grade, pathological type, tumor size, radiotherapy, and chemotherapy were significantly associated with OS (*p* < 0.05) (**Table 2**). Variables with *p* <0.10 from the univariable analysis were considered for multivariable analysis. The multivariable analysis showed that age, sex, primary tumor site, pathological type, tumor size, radiotherapy, chemotherapy, and thermal ablation were independently associated with OS (*p <*0.05). Regarding the pathological type, squamous cell carcinoma served as a reference, and adenocarcinoma (hazard ratio (HR) = 0.81, 95% CI: 0.69–0.95, *p* = 0.009) was shown to be a favorable prognostic factor for OS. However, large cell lung cancer (HR = 1.10, 95% CI: 0.68–1.77, *p* = 0.703) and the other pathological types (HR = 1.00, 95% CI: 0.64–1.56, *p* = 0.997) were not statistically related to OS. Compared with tumor size ≤3.0 cm, tumor sizes of 5.1–7.0 cm (HR = 1.31, 95% CI: 1.07–1.60, *p* = 0.009) and >7.0 cm (HR = 1.91, 95% CI: 1.52–2.40, *p <*0.001) were adverse prognostic factors for OS, and tumor sizes of 3.1–5.0 cm (HR = 1.12, 95% CI: 0.94–1.33, *p* = 0.203) did not significantly impact OS. The multivariable analysis of CSS showed that age, sex, primary tumor site, pathological type, tumor size, lymph node staging, radiotherapy, chemotherapy, and TA were independently associated with CSS (*p <*0.05) (**Table 3**). Overall, TA was associated with longer OS (*p* = 0.005) and CSS (*p* = 0.020).

**Table 2:** Univariate and multivariate analyses of OS after PSM.

Characteristics	Univariable		Multivariable	
	HR (95% CI)	*p* Value	HR (95% CI)	*p* Value	
Age (years)				
<70	1.00		1.00	
≥70	1.43 (1.26-1.63)	<0.001	1.21 (1.04-1.41)	0.014
Sex				
Male	1.00		1.00	
Female	0.73 (0.63-0.83)	<0.001	0.77 (0.67-0.89)	<0.001
Race				
White	1.00		1.00	
Black	0.91 (0.74-1.11)	0.346	0.89 (0.72-1.10)	0.285
Others	0.63 (0.39-1.00)	0.049	0.68 (0.43-1.09)	0.112
Laterality				
Left	1.00			
Right	1.02 (0.89-1.17)	0.779		
Tumor site				
Lung lobe	1.00		1.00	
Main bronchus	0.96 (0.79-1.16)	0.664	1.16 (0.95-1.43)	0.150
Overlapping lesion of lung	5.45 (2.25-13.22)	<0.001	2.91 (1.18-7.16)	0.020
Histological grade				
I/II	1.00			
III/IV	0.82 (0.69-0.98)	0.030	0.97 (0.81-1.17)	0.761
Unknown	0.96 (0.82-1.12)	0.606	0.96 (0.81-1.13)	0.603
Pathological type				
Squamous cell carcinoma	1.00			
Adenocarcinoma	0.81 (0.70-0.93)	0.004	0.81 (0.69-0.95)	0.009
Large cell lung cancer	0.82 (0.52-1.32)	0.419	1.10 (0.68-1.77)	0.703
Others	1.01 (0.65-1.56)	0.956	1.00 (0.64-1.56)	0.997
Tumor size				
≤3.0 cm	1.00			
3.1–5.0 cm	0.90 (0.75-1.06)	0.202	1.12 (0.94-1.33)	0.203
5.1–7.0 cm	1.02 (0.85-1.23)	0.796	1.31 (1.07-1.60)	0.009
>7.0 cm	1.29 (1.06-1.57)	0.012	1.91 (1.52-2.40)	<0.001
Lymph node staging				
N0	1.00			
N1	1.04 (0.83-1.31)	0.743		
N2	1.02 (0.88-1.19)	0.754		
N3	1.15 (0.90-1.46)	0.257		
Radiotherapy				
Yes	1.00			
No	1.61 (1.41-1.84)	<0.001	1.49 (1.26-1.78)	<0.001
Chemotherapy				
Yes	1.00			
No	1.71 (1.50-1.95)	<0.001	1.57 (1.31-1.89)	<0.001
Marital status				
Married				
Not married	0.92 (0.80-1.04)	0.187		
Thermal ablation				
Yes	1.00			
No	1.16 (1.00-1.35)	0.052	1.25 (1.07-1.46)	0.005

OS, overall survival; PSM, propensity score matching; HR, hazard ratio; CI, confidence interval.

**Table 3:** Univariate and multivariate analyses of CSS after PSM.

Characteristics	Univariable		Multivariable	
	HR (95% CI)	*p* Value	HR (95% CI)	*p* Value	
Age (years)				
<70	1.00		1.00	
≥70	1.30 (1.12-1.51)	<0.001	1.24 (1.05-1.46)	0.013
Sex				
Male	1.00		1.00	
Female	0.71 (0.61-0.83)	<0.001	0.78 (0.66-0.92)	0.002
Race				
White	1.00			
Black	0.92 (0.73-1.16)	0.473		
Others	0.69 (0.42-1.14)	0.145		
Laterality				
Left	1.00			
Right	1.01 (0.87-1.17)	0.902		
Tumor site				
Lung lobe	1.00		1.00	
Main bronchus	1.06 (0.87-1.31)	0.555	1.15 (0.92-1.44)	0.216
Overlapping lesion of lung	6.76 (2.78-16.41)	<0.001	3.08 (1.25-7.63)	0.015
Histological grade				
I/II	1.00			
III/IV	0.92 (0.75-1.12)	0.409		
Unknown	1.02 (0.86-1.23)	0.793		
Pathological type				
Squamous cell carcinoma	1.00		1.00	
Adenocarcinoma	0.75 (0.64-0.89)	0.001	0.78 (0.66-0.93)	0.006
Large cell lung cancer	0.73 (0.42-1.26)	0.260	1.03 (0.59-1.79)	0.924
Others	0.88 (0.53-1.47)	0.626	1.00 (0.59-1.70)	0.985
Tumor size				
≤3.0 cm	1.00		1.00	
3.1–5.0 cm	1.07 (0.89-1.29)	0.473	1.34 (1.10-1.63)	0.004
5.1–7.0 cm	1.27 (1.03-1.56)	0.023	1.61 (1.29-2.01)	<0.001
>7.0 cm	1.63 (1.31-2.02)	<0.001	2.22 (1.73-2.84)	<0.001
Lymph node staging				
N0	1.00		1.00	
N1	1.06 (0.81-1.37)	0.692	1.24 (0.95-1.63)	0.111
N2	1.18 (1.00-1.40)	0.046	1.54 (1.28-1.86)	<0.001
N3	1.32 (1.01-1.71)	0.041	1.69 (1.28-2.22)	<0.001
Radiotherapy				
Yes	1.00		1.00	
No	1.46 (1.26-1.70)	<0.001	1.62 (1.33-1.96)	<0.001
Chemotherapy				
Yes	1.00		1.00	
No	1.47 (1.27-1.70)	<0.001	1.60 (1.30-1.97)	<0.001
Marital status				
Married	1.00			
Not married	0.95 (0.82-1.10)	0.487		
Thermal ablation				
Yes	1.00		1.00	
No	1.21 (1.02-1.43)	0.003	1.23 (1.03-1.46)	0.020

CSS, cancer-specific survival; PSM, propensity score matching; HR, hazard ratio; CI, confidence interval.

### Subgroup analysis after PSM

**Figure 2:**
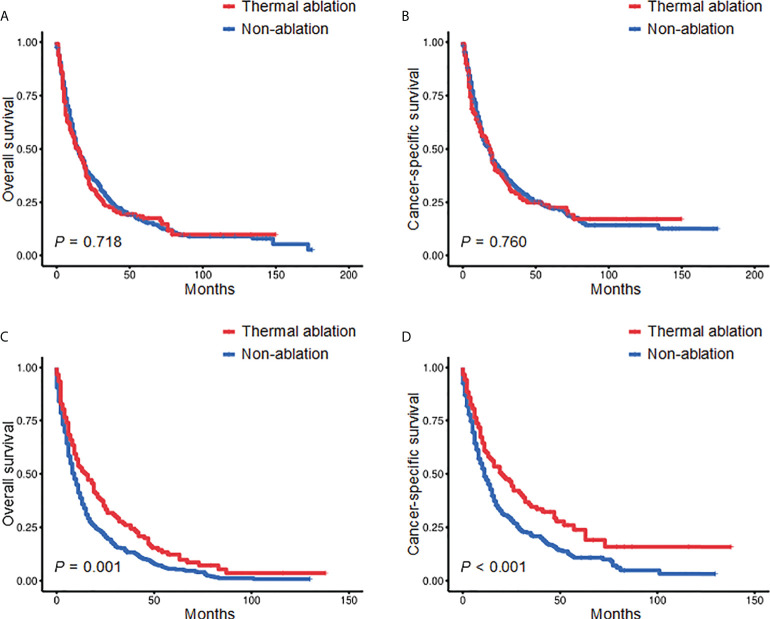
Kaplan-Meier survival curves comparing the TA and non-ablation groups when age was <70 years **(A, B)** or ≥70 years **(C, D)** after PSM. TA, thermal ablation; PSM, propensity score matching.

Kaplan-Meier was used to conduct subgroup analyses stratified by age and tumor size. When age was <70 years, there was no significant difference in the OS and CSS of the TA and non-ablation groups. The 3- and 5-year OS of the TA and non-ablation groups was 22.48% versus 25.95% and 17.40% versus 16.09%, respectively (*p* = 0.718, **Figure 2A**). The 3- and 5-year CSS of the TA and non-ablation groups was 29.07% versus 32.10% and 22.60% versus 22.51%, respectively (*p* = 0.760, **Figure 2B**). When age was ≥70 years, the TA group had longer OS and CSS than the non-ablation group. The 3- and 5-year OS of the TA and non-ablation groups was 26.02% versus 13.30% and 12.29% versus 5.61%, respectively (*p* = 0.001, **Figure 2C**). The 3- and 5-year CSS of the TA and non-ablation groups was 34.59% versus 20.82% and 23.84% versus 10.79%, respectively (*p* <0.001, **Figure 2D**).

**Figure 3:**
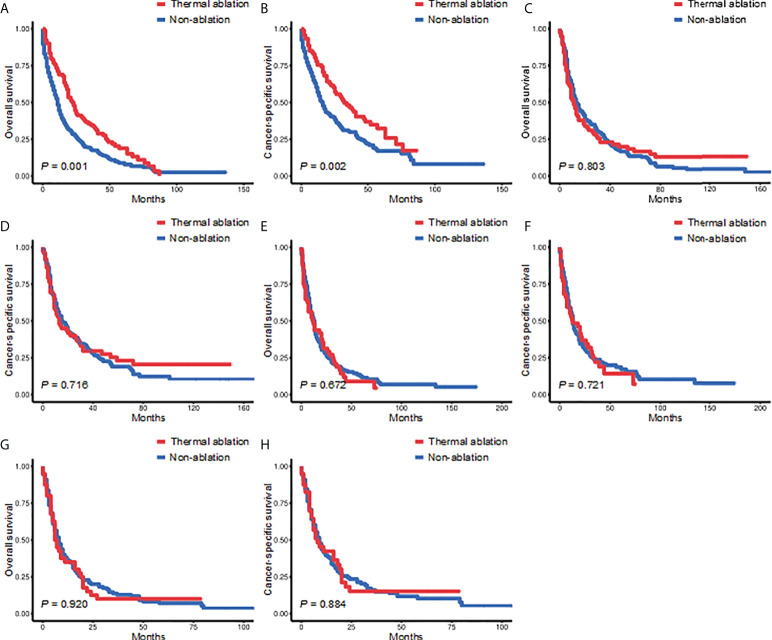
Kaplan-Meier survival curves for the TA and non-ablation groups when tumor size was ≤3.0 cm **(A, B)**, 3.1-5.0 cm **(C, D)**, 5.1-7.0 cm **(E, F)**, or >7.0 cm **(G, H)** after PSM. TA, thermal ablation; PSM, propensity score matching.

In patients with tumor size ≤3.0 cm, the 3- and 5-year OS of the TA and non-ablation groups was 34.44% versus 17.87% and 18.86% versus 8.89%, respectively (*p* = 0.001, **Figure 3A**). The 3- and 5-year CSS of the TA and non-ablation groups was 46.55% versus 29.85% and 32.35% versus 17.06%, respectively (*p* = 0.002, **Figure 3B**). However, no significant difference was observed in the OS and CSS of the TA and non-ablation groups for tumor sizes of 3.1–5.0 cm (3-year OS: 22.97% versus 24.77%, *p* = 0.803, **Figure 3C**; 3-year CSS: 29.57% versus 30.65%, *p* = 0.716, **Figure 3D**), 5.1–7.0 cm (3-year OS: 18.75% versus 18.99%, *p* = 0.672, **Figure 3E**; 3-year CSS: 22.12% versus 23.76%, *p* = 0.721, **Figure 3F**), and >7.0 cm (3-year OS: 10.00% versus 13.64%, *p* = 0.920, **Figure 3G**; 3-year CSS: 15.12% versus 15.98%, *p* = 0.884, **Figure 3H**).

**Figure 4:**
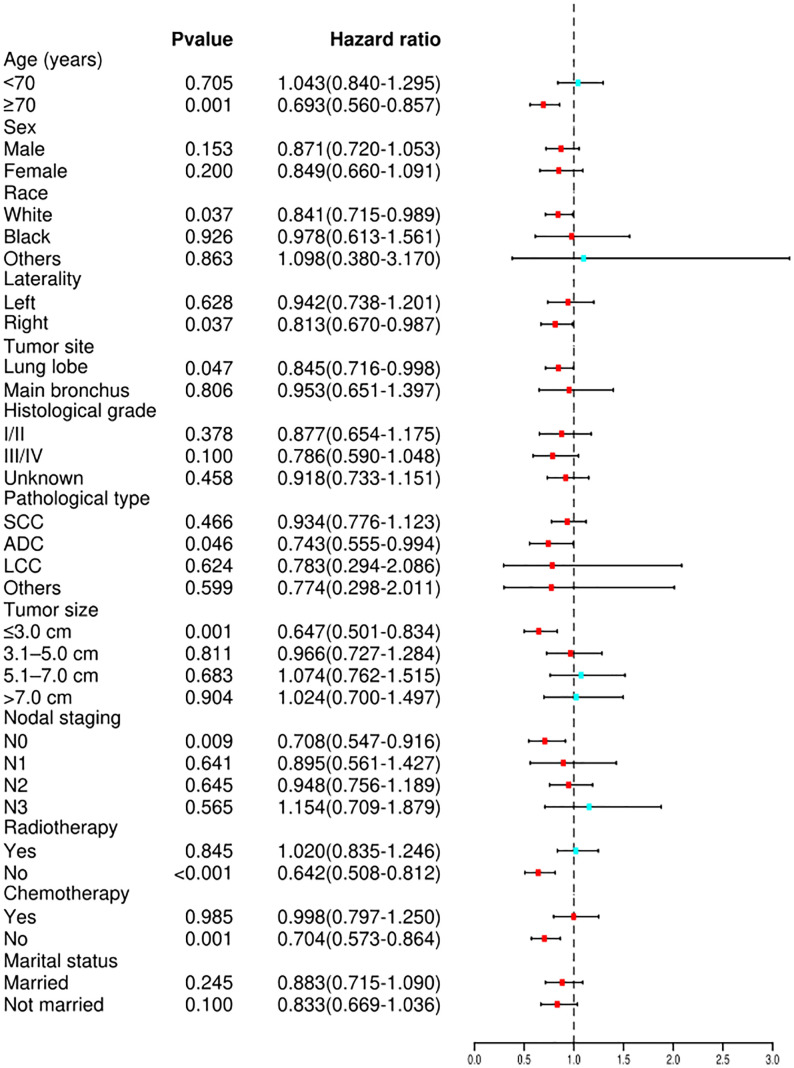
Forest plot depicting subgroup analysis of the OS between the TA and non-ablation groups after PSM. SCC, squamous cell carcinoma; ADC, adenocarcinoma; LCC, large cell carcinoma; OS, overall survival; TA, thermal ablation; PSM, propensity score matching.

**Figure 5:**
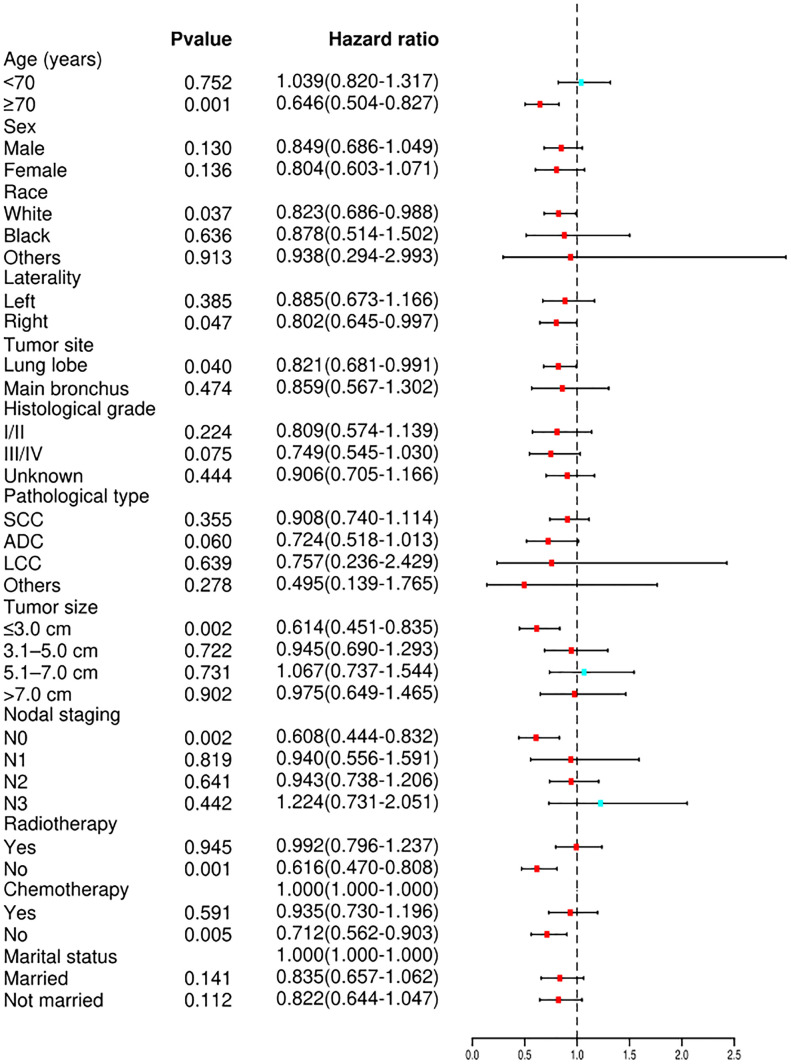
Forest plot depicting subgroup analysis of the CSS between the TA and non-ablation groups after PSM. SCC, squamous cell carcinoma; ADC, adenocarcinoma; LCC, large cell carcinoma; CSS, cancer-specific survival; TA, thermal ablation; PSM, propensity score matching.

Cox proportional hazard analyses were performed to explore the survival benefit of TA for patients in different subgroups. TA improved the OS of patients who were ≥70 years of age (HR = 0.693, 95% CI: 0.560–0.857, *p <*0.001) or white (HR = 0.841, 95% CI: 0.715–0.989, *p* = 0.037) and had right laterality (HR = 0.813, 95% CI: 0.670–0.987, *p* = 0.037), lung lobes (HR = 0.845, 95% CI: 0.716–0.998, *p* = 0.047), adenocarcinoma (HR = 0.743, 95% CI: 0.555–0.994, *p* = 0.046), tumor size ≤3.0 cm (HR = 0.647, 95% CI: 0.501–0.834, *p* = 0.001), N0 staging (HR = 0.708, 95% CI: 0.547–0.916, *p* = 0.009), non-radiotherapy (HR = 0.642, 95% CI: 0.508–0.812, *p <*0.001) and non-chemotherapy (HR = 0.704, 95% CI: 0.573–0.864, *p* = 0.001) (**Figure 4**). For CSS, subgroup analysis stratified by age, sex, race, histological grade, tumor site, laterality, lymph node staging, tumor size, radiotherapy, chemotherapy, and marital status gave similar results (**Figure 5**). A statistically improved OS (*p* = 0.046) but not CSS (*p* = 0.060) was observed in patients with TA when the pathology type was adenocarcinoma.

## Discussion

Data on 57,959 stage II-III NSCLC patients who were unsuitable for surgical resection were extracted from the SEER database. The findings revealed that TA improved the OS and CSS of stage II-III NSCLC patients who did not undergo surgical resection, particularly for those ≥70 years of age, with tumor size ≤3.0 cm, or who did not receive radiotherapy.

TA is considered a safe treatment for primary lung cancer (7, 20, 21). As an alternative therapy to surgical resection, TA causes irreversible damage to tumor cells using thermal energy (14, 31, 32). While previous studies have shown that TA is beneficial for stage I NSCLC patients (7, 18, 33), however, there is minimal data on the use of TA in stage II-III NSCLC patients. The current study found that TA could significantly improve the OS and CSS of stage II-III NSCLC patients without surgical resection. However, the effect of differences in clinicopathological features, such as age, race, tumor site, pathological type, tumor size, lymph node staging, and chemotherapy, cannot be overlooked. After PSM, baseline characteristics were similar between the TA and non-ablation groups in the adjusted cohorts and patients with TA still had longer OS and CSS than those without. This finding is consistent with results from a study by Heon et al. (34) which retrospectively evaluated the efficacy of CT-guided RFA in 77 NSCLC patients. Among stage I-II NSCLC patients, the median OS for those receiving RFA alone was 28.2 months, which was similar to patients receiving surgery alone (*p >*0.05). For stage III-IV NSCLC patients who received chemotherapy, the median OS of those receiving RFA was longer than the OS of those with no ablation (42 months versus 29 months, *p* = 0.03).

Multivariate Cox regression analysis showed that age, sex, tumor site, pathological type, tumor size, radiotherapy, chemotherapy, and thermal ablation were independently associated with the OS and CSS of stage II-III NSCLC patients without surgical resection. Subgroup analysis stratified by age showed that TA improved the OS and CSS of individuals ≥70 years of age with stage II-III NSCLC. When age was <70 years, no significant difference in OS or CSS was observed between the TA and non-ablation groups. These results were similar to a study on stage I NSCLC patients (35). Zeng et al. compared the efficacy of TA and wedge resection and found that the OS and CSS of stage I NSCLC patients who received TA and wedge resection were comparable for individuals >75 years of age (*p >*0.05). However, the OS and CSS were significantly shorter for patients who received TA than those who received wedge resection among individuals ≤75 years of age (*p <*0.05). This may be because older patients have more medical comorbidities and poorer performance status. As a minimally invasive treatment technique, TA benefits elderly patients by significantly decreasing the likelihood of complications (36, 37).

There was a statistically significant difference in the OS and CSS of patients receiving TA and those without when tumor size was ≤3.0 cm. However, when tumor size was >3.0 cm, no significant differences in OS and CSS were observed between the groups. Xu et al. retrospectively evaluated the efficacy of MWA in 234 NSCLC patients (38). The median OS was 35 months for patients with tumor size <3.0 cm and only 16 months for patients with tumor size ≥3.0 cm (*p <*0.001). This may be because of the limited amount of tumor necrosis caused by TA. The occurrence of incomplete ablation for patients with large tumors may increase the risk of leaving tumor remnants and disease recurrence.

Cox proportional hazard analyses were performed to distinguish between several groups that benefitted more from TA. TA improved the OS and CSS of patients who were white or had right laterality, lung lobes, N0 staging, non-radiotherapy, and non-chemotherapy (*p <*0.05). Steber et al. reported the long-term outcomes from a prospective single-arm, phase 2 study of 13 inoperable NSCLC patients receiving combined RFA and external beam radiotherapy (EBRT) (39). The median progression-free survival of patients with combined RFA and EBRT was 37.8 months, similar to the survival of patients who received EBRT alone. Thus, RFA combined with EBRT was not recommended for patients with NSCLC.

No statistically significant differences were observed in the OS and CSS of patients receiving TA or no ablation among those receiving chemotherapy. The advantage of TA was more pronounced in patients without chemotherapy (*p <*0.05). Wei et al. (40) reported that the median OS of Stage III-IV lung adenocarcinoma patients was not significantly different between groups receiving MWA combined with chemotherapy or chemotherapy alone (23.9 months versus 17.3 months, *p* = 0.140). A study of 49 NSCLC patients by Li et al. (41) also showed no statistical difference in 3‐year OS between those receiving MWA and chemotherapy or chemotherapy alone (21.057 months versus 17.843 months, *p >*0.05). In contrast, a study of 256 NSCLC patients by Xu et al. showed that the median OS was longer for patients with CT-guided RFA in combination with systemic chemotherapy than for those receiving chemotherapy alone (17.5 months versus 13.4 months, *p <*0.05) (42). Another retrospective study of 66 NSCLC patients reported by Feng et al. showed that patients who received MWA in combination with systemic chemotherapy had a longer median OS than those who received systemic chemotherapy alone (289.0 days versus 190.0 days, *p* = 0.018) (43). Overall, the effects of TA in combination with chemotherapy on OS and CSS remain unclear. Additional prospective multicenter randomized controlled trials are needed to explore the efficacy of TA in combination with chemotherapy in NSCLC patients.

Several factors may contribute to the survival benefits of TA among stage II-III NSCLC patients. First, TA can cause direct thermal injuries to targeted tissues. Temperatures exceeding 60°C can cause cellular membrane lysis, protein denaturation, and enzyme inactivation, which lead to rapid coagulative necrosis (15, 44). Second, in the tumor periphery beyond the border of immediate tissue coagulation, TA can cause indirect thermal injuries, including vascular injury, ischemia-reperfusion, release of lysosomal contents (16, 45, 46). Finally, TA may result in the release of abundant immunogenic intracellular substrates, which can initiate and upregulate steps in the cancer immunity cycle required to elicit an anti-cancer immune response (47, 48).

The current study has several limitations. First, there was a lack of detail in the SEER database on targeted therapy, immunotherapy, and the results of genetic testing. Targeted therapies can inhibit the protein products of aberrant genes. Currently, oncogenic aberrations in eight genes (EGFR, ALK, ROS1, BRAF, KRAS, NTRK, MET, and RET) are approved therapies for NSCLC (49). Immunotherapies have also proven efficacious in NSCLC patients (50). However, the status of targeted therapy or immunotherapy and the results of genetic testing were not evaluated as variables in this study so the effects of these factors on prognosis could not be explored. Second, the type of TA modality may impact the prognosis. However, the SEER database was unable to differentiate between TA modalities (e.g., RFA, MWA, and ultrasound ablation), so this information could not be included. Third, a large proportion of cases in the SEER database had no detailed information on tumor extension, such as the description of various organ invasions, so factors regarding the sith TNM stage could not be accurately transformed into the eighth TNM stage (subgroup, IIa, IIb, IIIa, IIIb, and IIIc) in stage II-III patients. Thus, stage II-III patients were not further stratified by stage, which may further bias the results. Finally, this study was retrospective so the strength of the results is weaker than it is for randomized controlled trials or prospective studies. Selection bias may be present because only patients with complete information were included.

## Conclusion

In summary, TA could be an effective alternative treatment for stage II-III NSCLC patients unsuitable for surgical resection, particularly those ≥70 years of age, with tumor size ≤3.0 cm, or who did not receive radiotherapy.

## Data availability statement

Publicly available data were analyzed in this study. The data can be found here: https://seer.cancer.gov/.

## Ethics statement

Ethical review and approval was not required for the study on human participants in accordance with the local legislation and institutional requirements. Written informed consent for participation was not required for this study in accordance with the national legislation and the institutional requirements.

## Author contributions

W-YY and FY designed the study. W-YY and YH collected the data. W-YY, QH, and MP analyzed and interpreted the data. W-YY, ZZ, and SX drafted the manuscript. MP and FY proofread the manuscript for important intellectual content. All authors contributed to the article and approved the submitted version.

## Funding

This work was supported by the National Natural Science Foundation of China (grant number 81972195), the Scientific Research Program of Hunan Provincial Health Commission (grant number 20201047), the Clinical Medical Technology Innovation Guide Project of Hunan Province (grant number 2020SK53408), and the National Clinical Key Specialty Construction Project.

## Acknowledgments

The authors thank all the staff and participants of the SEER program.

## Conflict of interest

The authors declare that the research was conducted in the absence of any commercial or financial relationships that could be construed as a potential conflict of interest.

## Publisher’s note

All claims expressed in this article are solely those of the authors and do not necessarily represent those of their affiliated organizations, or those of the publisher, the editors and the reviewers. Any product that may be evaluated in this article, or claim that may be made by its manufacturer, is not guaranteed or endorsed by the publisher.
